# From burr-hole to embolization: unveiling the evolution of chronic subdural hematoma research (2001–2025)

**DOI:** 10.3389/fneur.2026.1780300

**Published:** 2026-06-12

**Authors:** Minghao Man, Xingye Zhang, Shaochun Guo, Wei Chang, Yuan Wang, Bo Tian

**Affiliations:** Department of Neurosurgery, Tangdu Hospital, Airforce Medical University, Xi’an, China

**Keywords:** atorvastatin, bibliometric, chronic subdural hematoma, embolization, middle meningeal artery

## Abstract

**Objective:**

This study aims to perform a scientometric analysis of global research on chronic subdural hematoma (CSDH) spanning the years 2001 to 2025, with the goal of elucidating publication trends, geographic and institutional contributions, and emerging research frontiers.

**Methods:**

We analyzed 2,010 English articles from the Web of Science Core Collection using scientometric tools like CiteSpace, VOSviewer, and Scimago Graphica. Simultaneously, we reviewed clinical trials from PubMed to evaluate clinical progress. The analysis included annual publication counts, country and institution contributions, journal and reference co-citation networks, and keyword co-occurrence and burst detection.

**Results:**

The number of annual publications increased significantly from 40 publications (2000–2009) to 190 publications (2020–2025). The United States (22.4%), China (14.5%), and Japan (13.8%) emerged as the leading contributors. Prominent academic institutions included the University of Cambridge (62 publications), and Harvard University (42 publications). The research trajectory can be categorized into three distinct phases: an initial emphasis on the standardization of surgical procedures, specifically burr-hole drainage; a subsequent phase in the 2010s marked by pharmacological advancements, notably the efficacy of atorvastatin; and a recent focus on minimally invasive techniques, particularly middle meningeal artery embolization (MMAE).

**Conclusion:**

The management of CSDH has evolved towards integrated “drug-intervention-surgery” models, with MMAE and pharmacotherapy representing the forefront of current research. Future research priorities should include the exploration of genomic signatures, the development of biocompatible embolic materials, and the establishment of global multicenter collaborations to enhance precision management strategies.

## 1 Introduction

Chronic subdural hematoma (CSDH) is a prevalent neurosurgical condition marked by the progressive accumulation of blood between the dura mater and arachnoid membrane, primarily affecting the elderly population, with an incidence rate of 1 to 5.3 per 1,000 person-years. This condition is frequently associated with anticoagulation or antiplatelet therapy, cerebral atrophy, or minor head trauma (1). Despite advancements in diagnostic imaging modalities, such as high-resolution computed tomography (CT) and magnetic resonance imaging (MRI), as well as improvements in surgical techniques, CSDH continues to be a significant contributor to morbidity and mortality. Recurrence rates following initial treatment range from 10 to 30%, and case fatality rates can reach up to 12% in severe instances (1, 2). This substantial clinical burden highlights the necessity for a thorough understanding of research trends to inform evidence-based practice and foster innovation.

Over the past two decades, the management of CSDH has experienced significant paradigm shifts. Initial research concentrated on the standardization and efficacy of surgical procedures, with twist-drill craniotomy and burr-hole drainage being established as primary interventions. Nevertheless, challenges such as postoperative recurrence, infection, and cognitive impairment remained prevalent (3). The 2010s marked a period of transformative advancements: pharmacological treatments, such as atorvastatin, which targets inflammation and vascular remodeling, proved effective in reducing hematoma volume and recurrence (4). Additionally, minimally invasive techniques, including MMAE, emerged as viable alternatives to traditional surgery, especially for patients with recurrent or high-risk profiles (5). Furthermore, real-world data from extensive clinical trials, such as the MAGIC-MT study, have begun to redefine treatment protocols (6). Simultaneously, the advent of emerging technologies—such as radiomics, artificial intelligence-driven risk prediction, and omics-based biomarker discovery—has further enriched the landscape of translational research (7).

In light of the aforementioned context, a comprehensive evaluation of the research dynamics pertaining to CSDH is imperative. Although narrative reviews have encapsulated significant advancements in the field (8), there is a paucity of studies that have employed quantitative analyses to examine research trends, geographic distribution, or existing knowledge gaps through scientometric methodologies. Such analyses have the potential to uncover latent patterns in publication activity, pinpoint emerging research hotspots, and underscore areas that remain underexplored (9).

In this study, we employed scientometric methodologies to perform a comprehensive analysis of global research on CSDH from 2001 to 2025. Our objectives were to: (1) delineate temporal trends in publication output and research focus; (2) map geographic and institutional contributions along with collaborative networks; (3) identify high-impact journals, authors, and seminal studies; and (4) elucidate evolving research frontiers and hotspots. This research aims to provide a framework that will enable researchers and clinicians to prioritize future investigations and enhance CSDH management within the context of personalized medicine.

## 2 Materials and methods

### 2.1 Data extraction

This study employs a bibliometric approach to analyze metadata. On June 5, 2025, a systematic literature search was conducted using the Web of Science Core Collection (WoScc) database to identify studies on CSDH published between January 1, 2001, and May 30, 2025. The search criteria were limited to English-language articles, with a specific emphasis on CSDH across all pertinent fields to ensure comprehensive coverage. This included both research articles and review papers. Initially, a total of 2,022 articles were retrieved and screened based on their titles, keywords, abstracts, and full texts. Following the exclusion of irrelevant papers and the removal of duplicates, a final selection of 2,010 articles was made for further analysis.

WoScc is a multidisciplinary database, while PubMed, from the US National Library of Medicine, specializes in clinical research. We used PubMed alongside WoScc for a federated analysis, as it excels in aggregating clinical research data and accessing clinical trials. A systematic search for CSDH research in PubMed, using the Clinical Trial filter, identified 100 eligible studies, highlighting clinical research progress.

**Figure 1:**
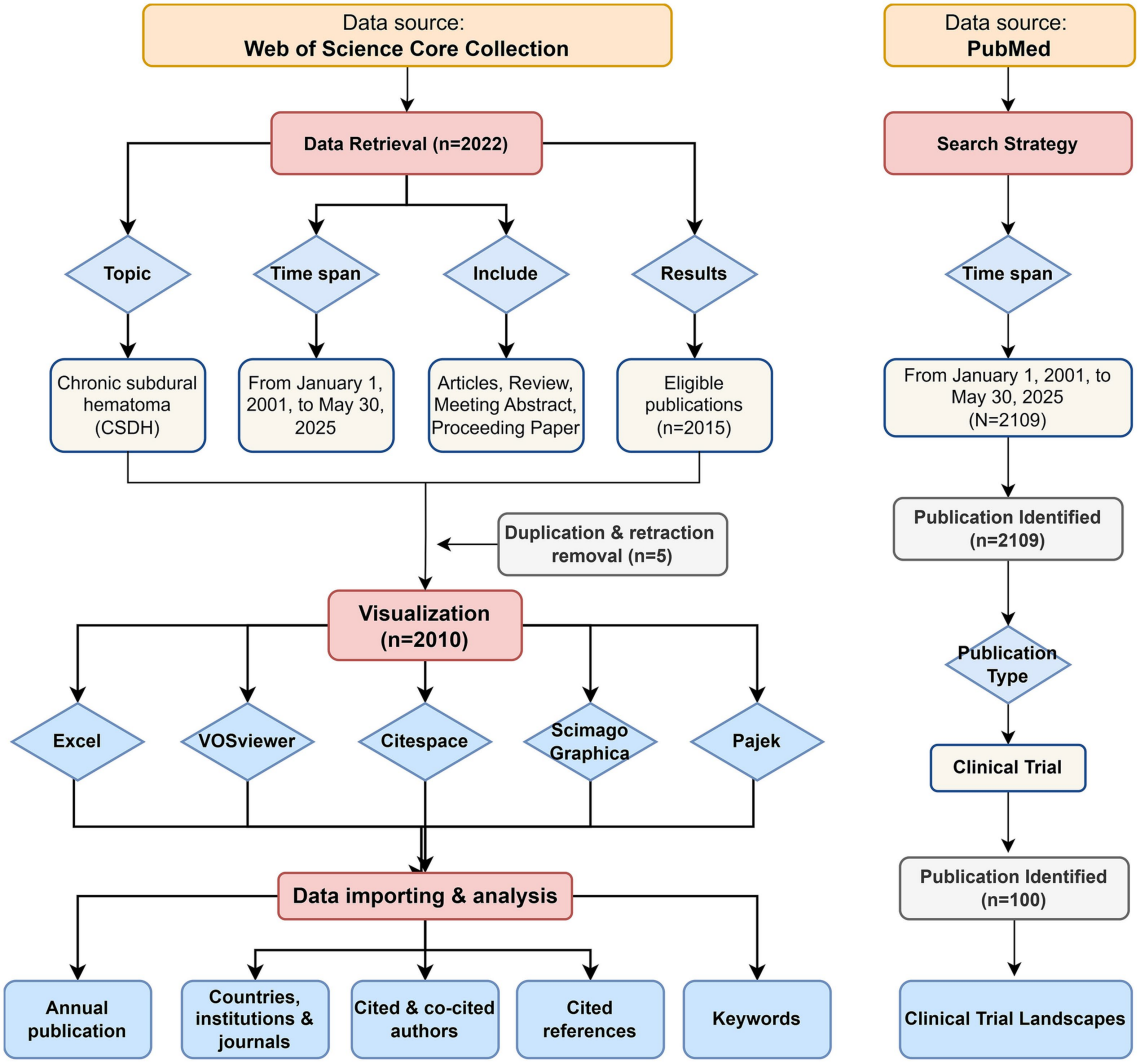
Literature search and data extraction process for bibliometric analysis.

The data searching strategies and retrieval formula for both WoScc and PubMed were presented in Supplementary File 1. The flowchart illustrating the literature selection process was presented in Figure 1. The original data that supports the conclusions of this article were presented as Supplementary File 2.

### 2.2 Analysis method

We performed scientometric and visualization analyses of the literature on CSDH utilizing CiteSpace (version 6.4.R1), VOSviewer (version 1.6.20), Scimago Graphica (1.0.51), and Pajek64 (version 6.01). Statistical analyses of the data were conducted using Microsoft Excel 2021. The analysis encompassed three primary aspects: (1) statistical analysis, which examined annual publication trends, countries/regions of publication, institutions, authors, cited authors, and journals; (2) co-citation cluster analysis of references to identify key research themes and track research trends; (3) keyword analysis to pinpoint research hotspots. CiteSpace, a bibliometric software package developed by Professor Chen, employs co-occurrence network mapping to visualize relationships within the literature (10). In CiteSpace, the size of a node corresponded to the frequency of co-occurrence, while the connections between nodes represented relational linkages, with the thickness of the lines indicating the strength of these relationships. The color of the node connections denoted different years. VOSviewer, an alternative bibliometric software developed by Leiden University, is characterized by its user-friendly interface and its ability to generate succinct graphical representations (11). In this study, the results were presented in both general view or heatmap, which were employed to analyze cited authors and keywords.

The analysis results of bar graph were generated using Scimago Graphica (1.0.51), the CNSknowall platform^1^ and ChiPlot,^2^ which were comprehensive web services for data analysis and visualization.

## 3 Results

### 3.1 Global publication and citation trend

**Figure 2:**
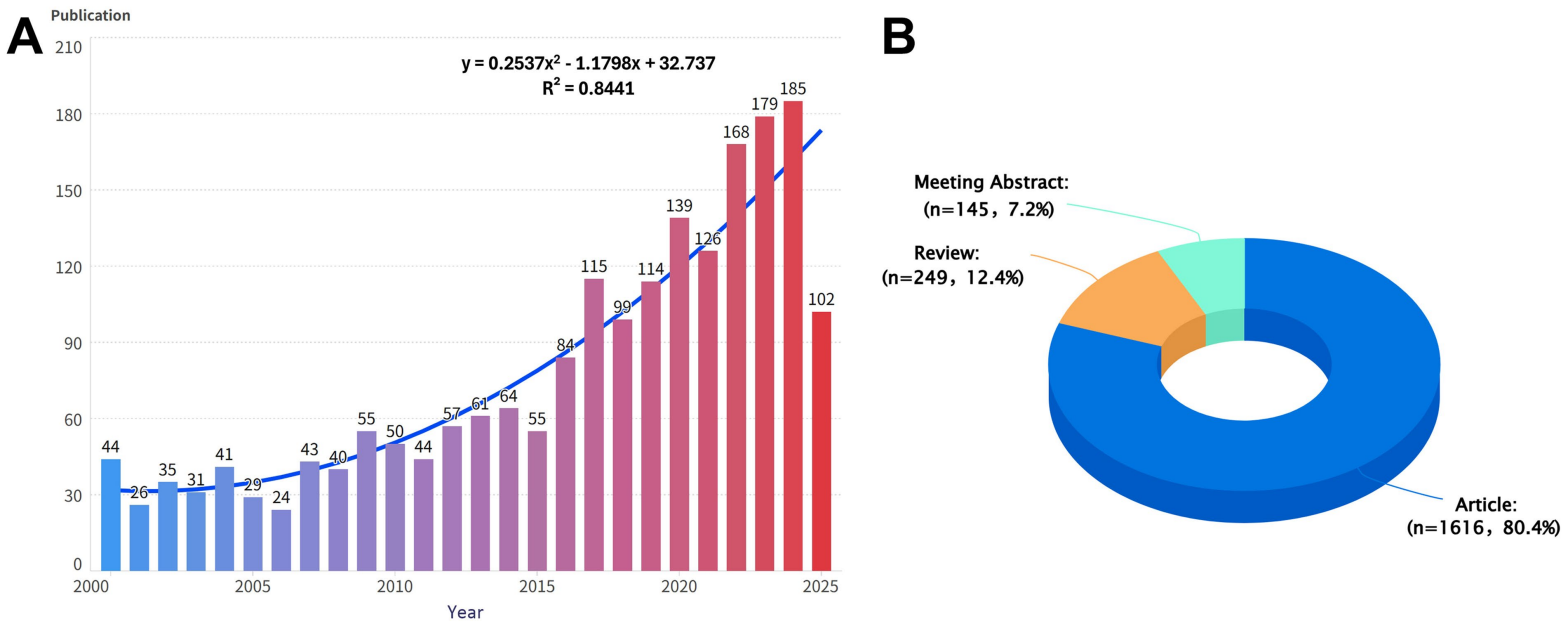
**(A)** The annual publication counts alongside a polynomial trend line, illustrating the trajectory of relevant literature from January 2001 to May 2025. **(B)** The categorical distribution of document types.

Based on the search strategy employed, we retrieved a total of 2,010 articles from the WoScc database, spanning the period from January 1, 2001, to May 31, 2025. As depicted in Figure 2A, there was a consistent annual increase in the number of research articles on CSDH, particularly notable since 2017, with annual publications exceeding 100. Figure 2B illustrated the specific categories and quantities of articles included in the study.

The annual publication volume concerning CSDH exhibited an overall upward trajectory, characterized by three distinct publication hotspots. From 2000 to 2009, the publication count remained around 40; between 2010 and 2019, there was a peak with publications reaching 120; and from 2020 to 2025, the publication count rose again, reaching 190. This trend was closely associated with the evolving research focus on CSDH, transitioning from traditional surgical approaches to investigations involving statin and MMAE therapies.

### 3.2 Country/region analysis

**Figure 3:**
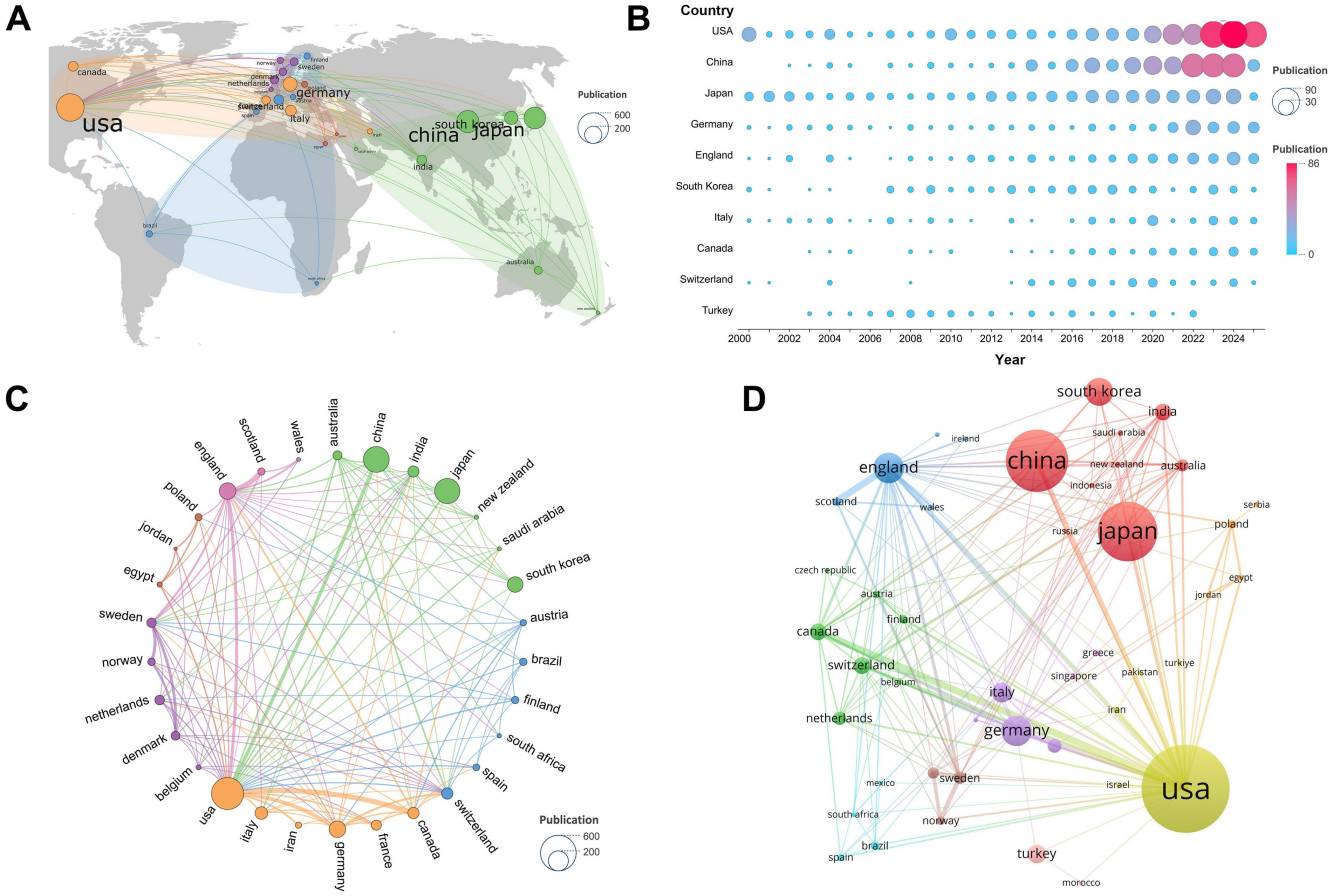
**(A)** The map delineates international collaborative efforts, depicting interconnections among countries, with the thickness of the lines representing the relative strength of these partnerships. **(B)** The trend analysis of the top 10 countries/regions with the highest annual publication counts. **(C)** A visualized chord diagram illustrating the collaborative networks among countries/regions. **(D)** The distribution of publications in CSDH research across various countries/regions.

A total of 84 countries and regions contributed to the research of CSDH, with their geographical distribution depicted in Figure 3A. The United States, China, and Japan emerged as the top three contributors in terms of the number of published articles. Figure 3B highlighted the top 10 countries based on the total number of publications and illustrated the annual distribution trends. The United States lead with 451 articles, accounting for 22.4% of the total, followed by China with 291 articles (14.5%), and Japan with 278 articles (13.8%) (Table 1). The United States also exceled in overall citation performance. Although China’s early contributions were limited, a marked increase in publications was observed since 2016. Figures 3C,D depicted international collaboration patterns, revealing that the United States engages in extensive partnerships with numerous countries, notably England, Canada, Germany, Sweden, and China.

**Table 1:** Top 20 countries ranked by number of publications on CSDH.

Rank	Country	Publication	Total citation	ACPP
1	USA	451	10,887	24.14
2	China	291	3,298	11.33
3	Japan	278	5,277	18.98
4	England	120	3,439	28.66
5	Germany	120	2,583	21.53
6	South Korea	106	2,236	21.09
7	Italy	70	1,268	18.11
8	Turkey	63	828	13.14
9	Canada	57	1,406	24.67
10	Switzerland	57	1,233	21.63
11	India	55	924	16.80
12	France	45	649	14.42
13	Netherlands	41	1,130	27.56
14	Sweden	39	1,034	26.51
15	Australia	36	936	26.00
16	Denmark	35	789	22.54
17	Norway	25	867	34.68
18	Brazil	24	348	14.50
19	Finland	24	583	24.29
20	Scotland	23	586	25.48

ACPP, average citations per paper; CSDH: chronic subdural hematoma.

### 3.3 Institutional analysis

**Figure 4:**
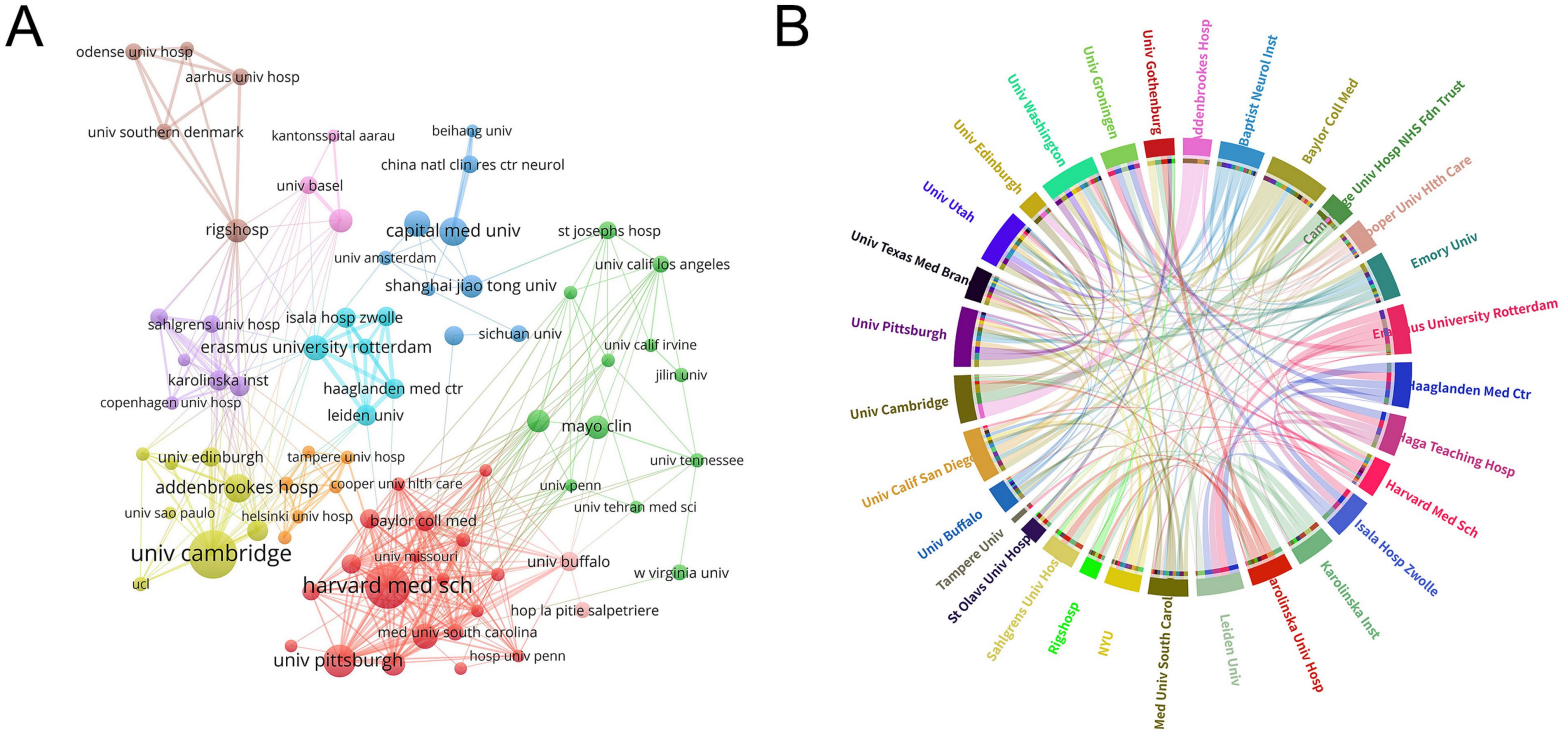
**(A)** Institutional collaboration network. Each node signifies an institution, with node size proportional to the institution’s publication output. The connecting lines between nodes indicate collaborative relationships between institutions. **(B)** A visualized chord diagram illustrating institutional collaboration.

A total of 234 institutions contributed research papers pertaining to CSDH. Table 2 presented the top 15 institutions ranked by the number of publications. Among these, the University of Cambridge and its affiliated institutions produced the highest number of papers, totaling 62 publications. Harvard University closely follows with 42 articles. The University of Pittsburgh and Addenbrooke’s Hospital published 28 and 23 articles, respectively. In terms of citations, the cumulative citation count for articles from the University of Cambridge system and Harvard University was significantly higher than others, with totals of 2,247 and 1,113, respectively. This underscored the substantial impact these institutions had in the CSDH research field. Figure 4A provided a visual representation of collaborative networks among countries, regions, and institutions, where each node denoted an institution. The size of each node reflected both the number of publications and the institution’s centrality within the network. The institutional co-authorship analysis conducted using VOSviewer further corroborated these dominant relationships (Figure 4B). The University of Cambridge exhibited strong collaborative ties with other European centers, while Harvard University maintained numerous partnerships with American universities hospitals and other institutions.

**Table 2:** Top 15 institutions ranked by publications on CSDH.

Rank	Institution	Publication	Total citation	ACPP
1	University of Cambridge	62	2,247	36.24
2	Harvard Medical School	42	1,113	26.50
3	University of Pittsburgh	28	538	19.21
4	Addenbrooke’s Hospital	23	824	35.83
5	Capital Medical University	23	159	6.91
6	Zhejiang University	21	287	13.67
7	Erasmus University Rotterdam	20	504	25.20
8	University of Washington	20	575	28.75
9	Mayo clinic	18	256	14.22
10	Copenhagen University Hospital Rigshospitalet	18	358	19.89
11	The University Hospital Basel	18	217	12.06
12	The University of Utah	18	481	26.72
13	Shanghai Jiao Tong University	17	270	15.88
14	University of Toronto	17	294	17.29
15	Leiden University	16	352	22.00

ACPP, average citations per paper; CSDH: chronic subdural hematoma.

### 3.4 Analysis of journals

**Figure 5:**
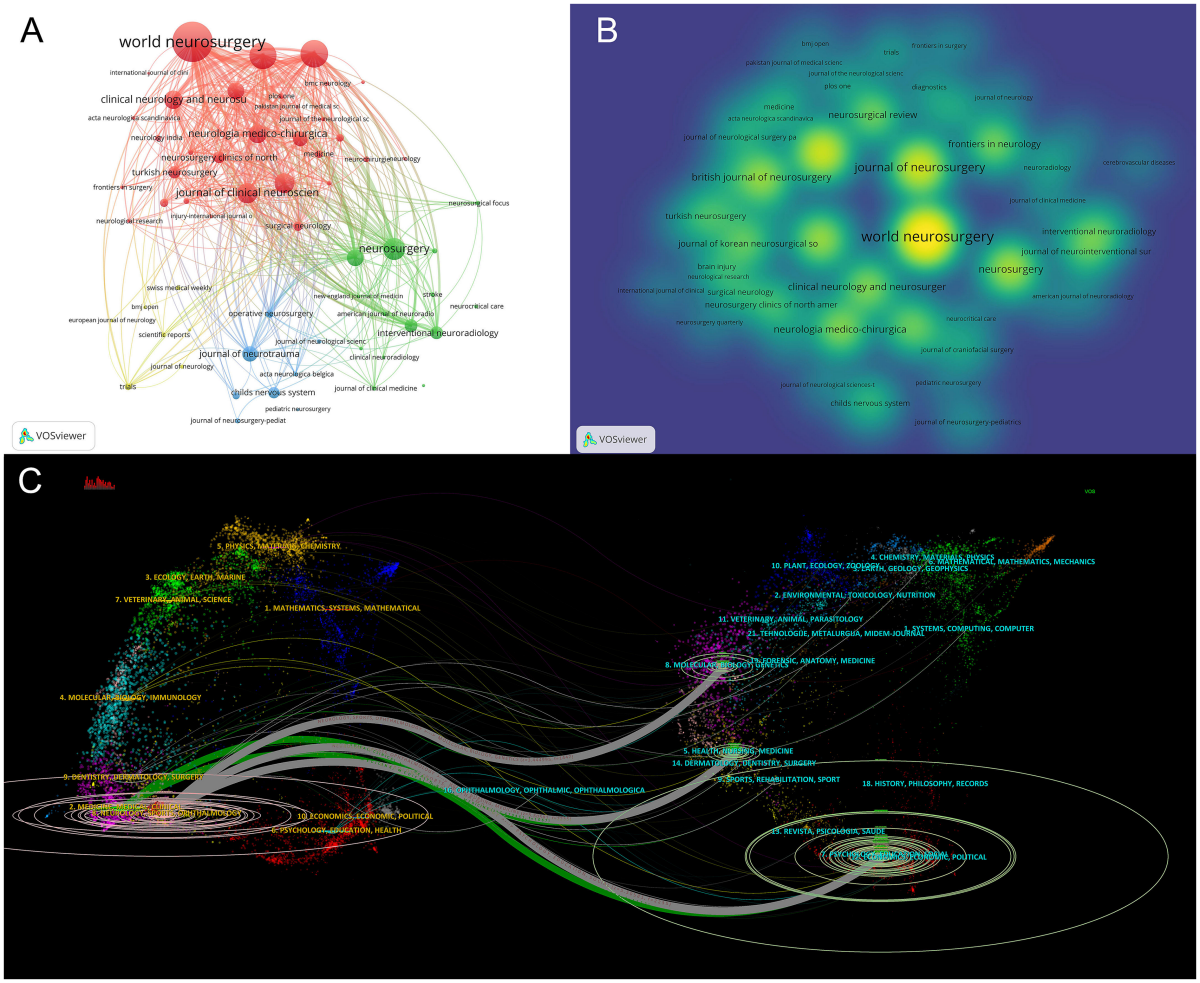
**(A)** Journals co-citation analysis. **(B)** Density map of journals. **(C)** A dual-map overlay of journals on CSDH.

Over the past 25 years, a total of 272 journals have published studies pertinent to CSDH. Based on the frequency of published articles, the top five journals were as follows: *World Neurosurgery* (201 articles), *Journal of Neurosurgery* (112 articles), *Acta Neurochirurgica* (109 articles), *Neurosurgery* (82 articles), and *Journal of Clinical Neuroscience* (74 articles) (Table 3). We ultimately identified 65 journals that were cited at least 80 times and constructed a co-citation network (Figures 5A,B). The journals with the highest co-citation counts were *Journal of Neurosurgery* (5,641 citations), *Neurosurgery* (2,661 citations), and *Acta Neurochirurgica* (2,626 citations), respectively.

**Table 3:** Top 10 journals in terms of the number of published papers.

Rank	Journals	Publication	Citation	IF	JCR quartile
1	World Neurosurgery	201	2,993	2.1	Q2
2	Journal of Neurosurgery	112	4,762	3.6	Q1
3	Acta Neurochirurgica	109	2,368	1.9	Q2
4	Neurosurgery	82	1,722	3.9	Q1
5	Journal of Clinical Neuroscience	74	1,680	1.8	Q3
6	British Journal of Neurosurgery	70	1,282	0.8	Q3
7	Neurologia Medico-Chirurgica	63	1,037	2.3	Q2
8	Clinical Neurology and Neurosurgery	62	1,035	1.6	Q2
9	Frontiers in Neurology	56	442	2.8	Q2
10	Neurosurgical Review	54	1,324	2.5	Q1

TC, total citation; IF, impact factor; JCR, journal citation reports.

The dual-map overlay of journals served as a valuable tool for depicting the distribution of subjects across academic journals, the evolution of citation trajectories, and the relocation of research centers. Research on CSDH spanned a wide array of disciplines and fields. Figure 5C illustrated a dual-map overlay of journals related to CSDH. The clusters on the left depicted the collection of citing journals, representing the current state of knowledge, while the clusters on the right depicted the collection of cited journals, demonstrating the knowledge base currently referenced by the forefront of research. The length of each ellipse corresponded to the number of authors, and the width corresponded to the number of publications. The curves connecting the two sides signified citation relationships, with different colors distinguishing subject areas. The thickness of the curve indicated the strength of the connection.

As depicted in the figure, the citing journals were predominantly distributed across discipline #2 (Medicine, Medical, Clinical) and #8 (Neurology, Sports, Ophthalmology). In contrast, the cited journals were primarily concentrated within discipline #7 (Psychology, Education, Social). Furthermore, a portion of the cited journals also spanned discipline #5 (Health, Nursing, Medicine) and #8 (Molecular, Biology, Genetics). Notably, the literature within discipline #8 (Neurology, Sports, Ophthalmology) was significantly influenced by discipline #7 (Psychology, Education, Social), as indicated by the statistical values (*z* = 5.05, *f* = 2,235).

### 3.5 Author analysis

A total of 5,933 authors contributed to research within the field of CSDH. Among these, Christopher S. Ogilvy was identified as the most prolific author, having published 24 articles, which constituted 1.2% of the total publications. He was closely followed by Koji Osuka, with 22 publications (1.1%), and Kare Fugleholm, with 20 publications (1%). Table 4 presented the top 10 authors ranked by publication volume. Collectively, these 10 authors produced 184 articles, representing 9.2% of the total articles. Regarding citation metrics, Peter J. Hutchinson was the most cited author, with 1,125 citations, followed by Angelos G. Kolias, with 650 citations, and Ruben Dammers, with 517 citations (Table 4).

**Table 4:** Top 20 authors ranked by publication in the research of CSDH.

Rank	Authors	Publication	Citation	ACPP
1	Ogilvy, Christopher S.	24	372	15.50
2	Osuka, Koji	22	174	7.91
3	Fugleholm, Kare	20	218	10.90
4	Dammers, Ruben	19	517	27.21
5	Hutchinson, Peter J.	18	1,125	62.50
6	Takayasu, Masakazu	17	151	8.88
7	Jellema, Korne	16	453	28.31
8	Jensen, Thorbjorn Soren Ronn	16	131	8.19
9	Kan, Peter	16	325	20.31
10	Lingsma, Hester F.	16	476	29.75
11	Soleman, Jehuda	16	287	17.94
12	Thomas, Ajith J.	16	347	21.69
13	van der Gaag, Niels A.	16	420	26.25
14	den Hertog, Heleen M.	14	410	29.29
15	Jiang, Rongcai	14	207	14.79
16	Kolias, Angelos G.	14	653	46.64
17	Liu, Weiming	14	92	6.57
18	Ou, Yunwei	14	92	6.57
19	Zhang, Jianning	14	239	17.07
20	Aoyama, Masahiro	13	117	9.00

ACPP, average citations per paper; CSDH: chronic subdural hematoma.

**Figure 6:**
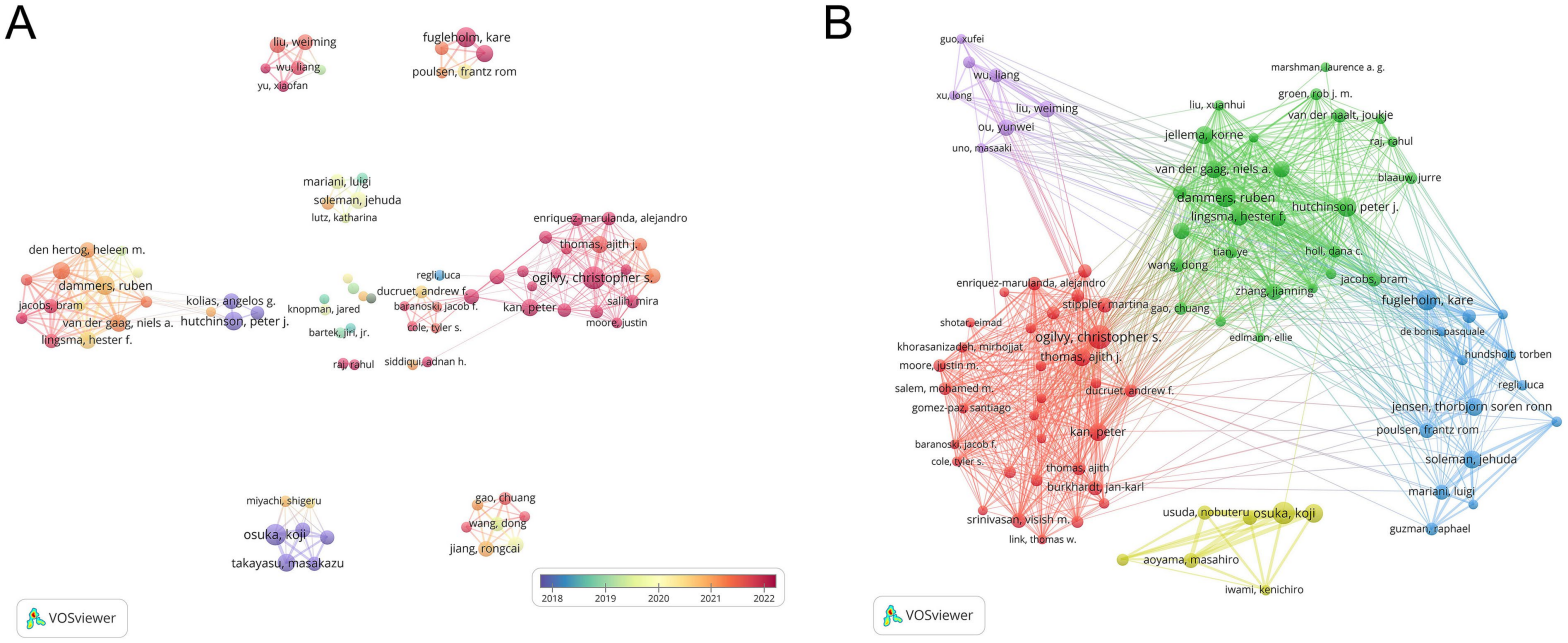
Visual representation of the author collaboration network. **(A)** Collaboration graph depicting authors with more than seven publications. **(B)** Collaboration graph illustrates authors with over 80 citations. The size of the nodes and fonts is directly proportional to the number of articles published, while the thickness of the connecting lines indicates the intensity of collaboration between authors.

The successful execution of a research project frequently hinged on the collaborative efforts of multiple researchers. By conducting an in-depth analysis of collaborative networks among authors, we can identify pivotal researchers within a specific academic domain and evaluate the extent of their collaboration. We provided a visual depiction of the author collaboration network. Specifically, Figure 6A highlighted the 87 researchers who published more than seven papers, whereas Figure 6B illustrated the collaboration graph of 76 authors who had been cited over 80 times. Our study indicated that the collaborative relationships among these authors demonstrated a distinct clustered distribution, which may be directly associated with shifts in treatment methodologies and research focal points, as inferred from the content and publication timelines of the relevant authors’ works. For instance, Jiang, Rongcai, and colleagues primarily concentrated on the treatment of atorvastatin, while Ogilvy, Christopher, and associates advanced the treatment and development of MMAE.

### 3.6 Co-citation analysis of reference

**Figure 7:**
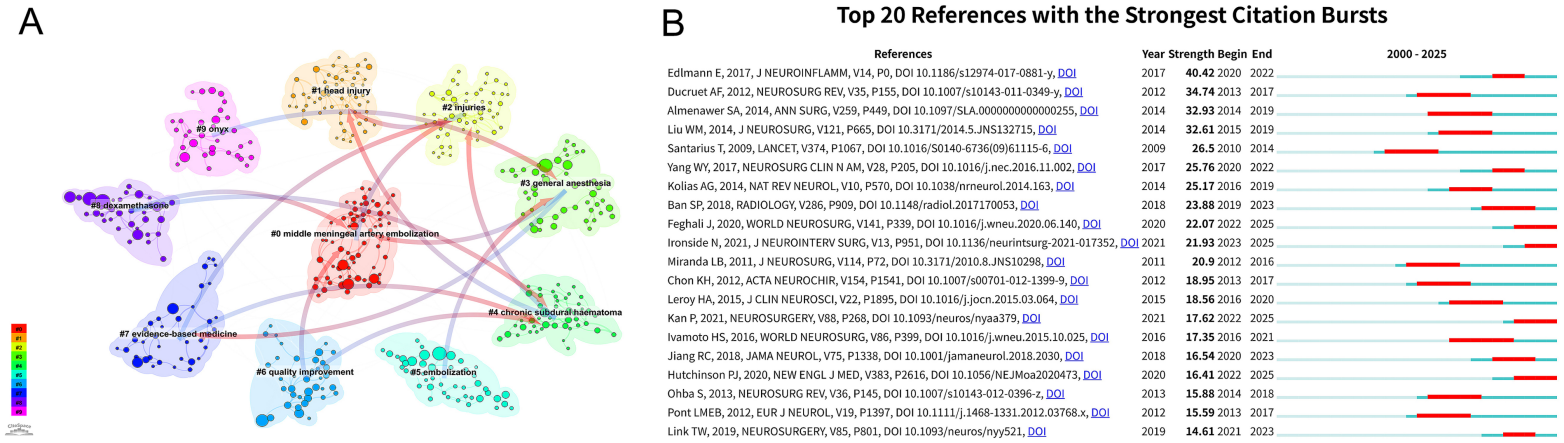
**(A)** Visualized cluster network analysis of CSDH research publications with 10 largest citation clusters. **(B)** Top 20 references with the strongest citation bursts.

The 10 most frequently cited references were comprehensively listed in Table 5. These highly cited articles were published in prestigious journals, including *The Lancet* (Impact Factor, IF = 88.5), *Journal of Neurosurgery* (IF = 3.6), *Journal of Neurotrauma* (IF = 3.8), and *Neurosurgery* (IF = 3.9). Notably, the article authored by Santarius and published in *The Lancet* in 2009 emerged as the most co-cited reference within CSDH research field, accumulating a total of 333 co-citations. A total of 183 references, each with a minimum of 35 citations, were ultimately included to conduct a reference co-citation analysis (Figure 7). Similar to the author’s collaborative network, the reference co-citation network also demonstrated significant clustered distribution pattern.

**Table 5:** Top 20 authors ranked by co-citation in the research of CSDH.

Rank	Authors	Co-citation
1	Hutchinson, Peter J.	731
2	Ogilvy, Christopher S.	675
3	Dammers, Ruben	656
4	Santarius, Thomas	603
5	Jellema, Korne	573
6	Lingsma, Hester F.	554
7	van der Gaag, Niels A.	541
8	Thomas, Ajith J.	521
9	Kan, Peter	505
10	Kolias, Angelos G.	492
11	den Hertog, Heleen M.	490
12	Osuka, Koji	466
13	Ducruet, Andrew F.	458
14	Fugleholm, Kare	436
15	Zhang, Jianning	417
16	Watanabe, Yasuo	415
17	Poulsen, Frantz Rom	412
18	Jiang, Rongcai	408
19	Dirven, Clemens M. F.	407
20	Takayasu, Masakazu	404

CSDH, chronic subdural hematoma.

To further examine the characteristics of references, we performed a cluster analysis. Figure 7A depicted the CiteSpace visualization of the co-cited literature cluster network, highlighting 10 largest citation clusters. The figure revealed that CSDH research primarily concentrated on key topics, such as the middle meningeal artery embolization (#0), which was currently the most prominent and extensively discussed topic in CSDH treatment. Other significant areas of focus included the causes and mechanisms of injury (#1 head injury, #2 injury), treatment methodologies (#5 embolization, #8 dexamethasone, and #9 Onyx), and prognosis evaluation (#6 quality improvement).

A burst in citation frequency was characterized by a rapid and substantial increase in the number of citations a document accrues over a given period, surpassing the document’s average citation rate. Analyzing such bursts can facilitate understanding shifts in research focus over time, and aid in examining prevailing trends and research interests within a particular academic domain. Figure 7B depicted the 20 most frequently cited references. In this figure, red bars denoted high citation frequencies, whereas blue bars indicated lower citation frequencies. Notably, among the most cited articles, the reference exhibiting the highest intensity of citation burst (intensity = 40.42, burst period = 2020–2022) was the article authored by Eldmann E. et al., published in 2017 in the *Journal of Neuroinflammation*, which ranked 8th among the most cited articles.

### 3.7 Keyword analysis

**Figure 8:**
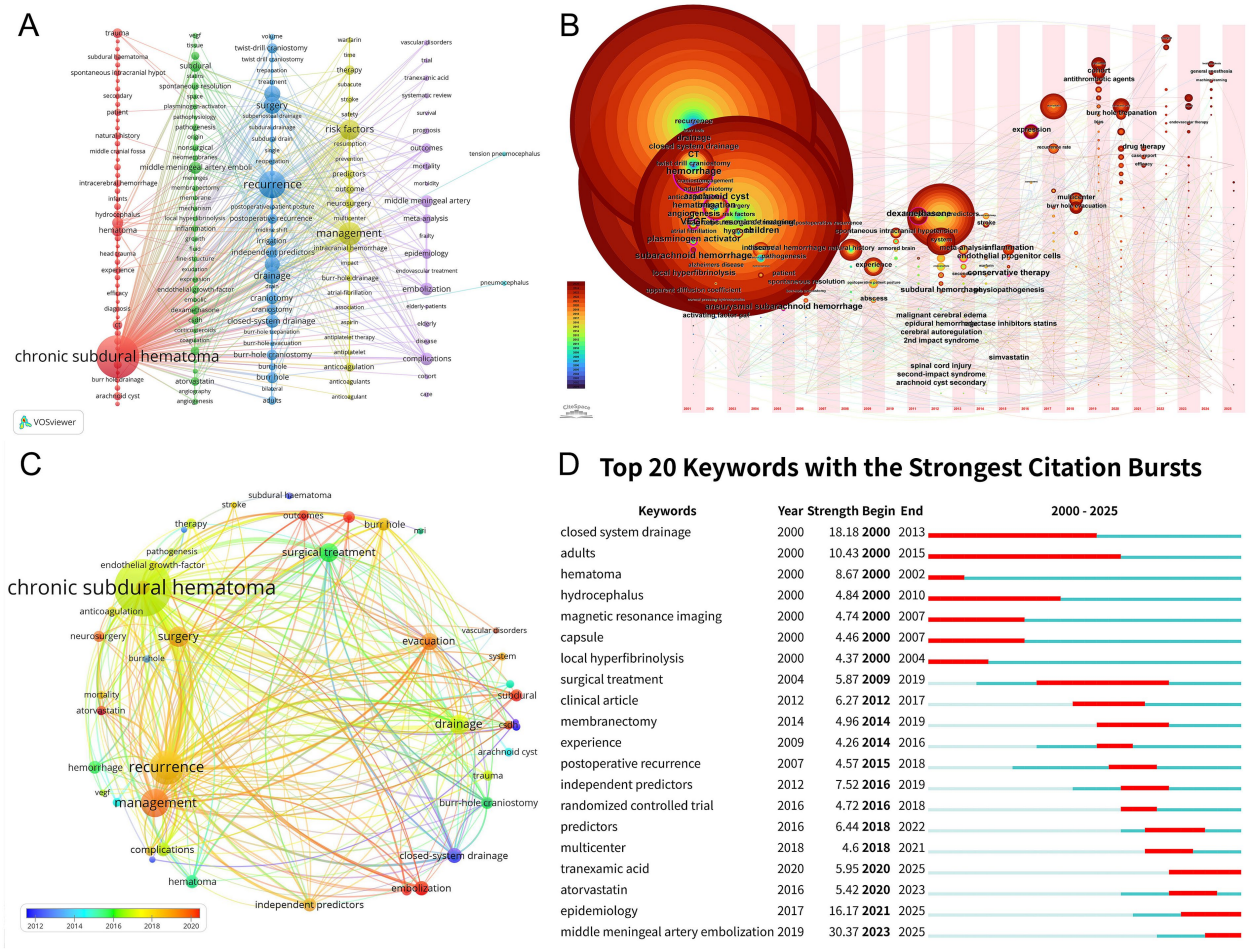
Network visualization of keyword co-occurrence. **(A)** Cluster network graph. **(B)** Keyword time-zone map. Circle size on the timeline reflects citation frequency and links show co-citation relationships, with node position indicating citation years. **(C)** The evolution and interrelation of popular research topics. **(D)** The top 20 keywords with the highest citation burst rates.

A total of 4,586 keywords were extracted. Table 6 presented the top 20 keywords with the highest co-occurrence frequencies. Eighty-four keywords with a minimum 30 co-occurrence frequency were selected to construct network graphs for co-occurrence analysis (Figure 8A), which consisted of six clusters. Cluster 1 (red) primarily addressed fundamental research related to the clinical presentation of CSDH, including aspects such as hydrocephalus and its natural history. Cluster 2 (green) focused on pathogenesis and recent advancements in treatment methods, including MMAE, atorvastatin, and endothelial growth factors. Cluster 3 (blue) highlighted traditional surgical treatment approaches, such as drilling and subdural drainage. Cluster 4 (yellow) was concerned with prognosis and perioperative management, encompassing risk factors, management strategies, and anticoagulation. Cluster 5 (purple) emphasized the management of complications, including mortality and outcomes (see Table 7).

**Table 6:** Top 10 references with highest citations in the research of CSDH.

Article title	First author	Journal	Citations	Publication year	Core content
Use of drains versus no drains after burr-hole evacuation of chronic subdural haematoma: a randomised controlled trial	Thomas Santarius	Lancet	333	2009	The implementation of a drainage system following burr-hole evacuation of chronic subdural hematoma is considered safe and is correlated with decreased recurrence rates and mortality within a six-month period
Outcome of contemporary surgery for chronic subdural haematoma: evidence based review	R. Weigel	Journal of Neurology, Neurosurgery and Psychiatry	285	2003	The main techniques for treating chronic subdural haematoma in modern neurosurgery—twist drill craniostomy, burr hole craniostomy, and craniotomy—vary in morbidity, mortality, recurrence, and cure rates. Twist drill and burr hole craniostomy are primary treatments, while craniotomy serves as a secondary option
Pathophysiology of chronic subdural haematoma: inflammation, angiogenesis and implications for pharmacotherapy	Ellie Edlmann	Journal of Neuroinflammation	273	2017	This review examines several critical processes involved in the development of CSDH, specifically angiogenesis, fibrinolysis, and inflammation
Factors in the natural history of chronic subdural hematomas that influence their postoperative recurrence	H. Nakaguchi	Journal of Neurosurgery	231	2001	Classifying CSDH based on their internal architecture and intracranial extension could be beneficial in predicting the likelihood of postoperative recurrence
The surgical management of chronic subdural hematoma	Andrew F. Ducruet	Neurosurgical Review	227	2012	This review summarized the epidemiology and pathophysiology of CSDH and address controversial management topics, such as post-operative anticoagulant resumption timing, anti-epileptic prophylaxis effectiveness, mobilization protocols after CSDH evacuation, and the effectiveness of different surgical techniques
Chronic subdural hematoma management: a systematic review and meta-analysis of 34,829 patients	Saleh A. Almenawer	Annals of Surgery	223	2014	Percutaneous bedside twist-drill drainage represents a relatively safe and effective initial management strategy. These findings have the potential to reduce healthcare costs and mitigate perioperative risks associated with general anesthesia
Surgical treatment of chronic subdural hematoma in 500 consecutive cases: clinical characteristics, surgical outcome, complications, and recurrence rate	K. Mori	Neurologia Medico-Chirurgica	222	2001	This study assessed the clinical characteristics, radiological findings, and surgical outcomes in a substantial cohort of patients treated at a single institution
Chronic subdural haematoma: modern management and emerging therapies	Angelos G. Kolias	Nature Reviews Neurology	212	2014	This review offers a comprehensive examination of the current management strategies for CSDH and discusses potential future methodologies that may enhance patient care and outcomes
Chronic subdural hematoma in the elderly: not a benign disease	Lucas Bernardes Miranda	Journal of Neurosurgery	180	2011	In this initial report on the long-term outcomes of elderly patients with CSDH, the authors identified sustained excess mortality extending up to one-year post-diagnosis. These findings challenge the perception of CSDH as a benign condition and suggest that it may serve as an indicator of other underlying chronic diseases, akin to hip fractures
Actual and projected incidence rates for chronic subdural hematomas in United States Veterans Administration and civilian populations	David Balser	Journal of Neurosurgery	178	2015	The incidence of CSDH is increasing, and it is anticipated that by 2030, CSDH will become the most prevalent cranial neurosurgical condition among adults

CSDH, chronic subdural hematoma.

**Table 7:** Top 20 keywords in the research of CSDH.

Rank	Keywords	Occurrences
1	Chronic subdural hematoma	1,315
2	Recurrence	526
3	Management	375
4	Risk factors	289
5	Surgery	212
6	Drainage	200
7	Surgical treatment	182
8	Evacuation	157
9	Closed-system drainage	122
10	Craniotomy	116
11	Embolization	113
12	Complications	109
13	Middle meningeal artery	108
14	Middle meningeal artery embolization	104
15	Independent predictors	99
16	Hematoma	97
17	Burr hole	91
18	Hemorrhage	90
19	Subdural	88
20	Burr-hole craniostomy	82

CSDH, chronic subdural hematoma.

By employing time as the ordinate, we identified temporal nodes and durations associated with each keyword, resulting in the creation of a keyword time-zone map (Figure 8B). The research focus has transitioned from traditional surgical methods, such as burr-hole drainage, to the prediction of risk factors and the application of embolization techniques. As illustrated, a significant number of high-frequency, persistent keywords emerged around 2002, including “closed system drainage,” “twist drill craniotomy,” “plasminogen activator,” and “pathogenesis.” Between 2009 and 2015, several high-frequency, enduring keywords became prominent, such as “dexamethasone,” “statins,” and “conservative therapy.” Since 2018, the prevalent keywords gradually shifted towards “drug therapy,” “antithrombotic agents,” “multicenter,” and “endovascular therapy.” Figure 8C further demonstrated the evolution and interrelationships of research hotspots in CSDH over time.

Figure 8D presented the top 20 keywords exhibiting the most significant citation bursts. Among these, “MMAE” demonstrated the highest burst intensity (30.37), followed by “closed system drainage” (18.18), “epidemiology” (16.17), “adults” (10.43), and “hematoma” (8.67). Analyzing the temporal evolution of these keywords revealed a gradual transition in research hotspots and a continual evolution of treatment concepts.

### 3.8 Clinical progress

A review of 100 clinical trials from PubMed highlighted key findings on CSDH treatment. Randomized studies favored single burr-hole drainage over double (12–14) and preferred burr-hole craniostomy to twist-drill or minicraniotomy (15, 16). Postoperative subperiosteal drainage reduced recurrence rates, especially in patients on anticoagulants (17–21). The DRAIN TIME 2 trial demonstrated that short drainage duration (24–48 h) was non-inferior to longer durations in preventing recurrence (22).

Adjunctive strategies to reduce recurrence have gained prominence. MMAE, evaluated in multiple high-quality randomized controlled trials (RCTs), significantly lowered recurrence when combined with surgery (6, 23–26). Pharmacologic approaches also showed promise: postoperative tranexamic acid reduced recurrence without increasing complications (27, 28); atorvastatin demonstrated efficacy in Chinese populations (4); and the Japanese Kampo medicine Goreisan exhibited preventive effects in prospective trials (29, 30).

Perioperative management has been refined through rigorous investigation. The GET-UP trial established that early mobilization (within 48 h) improved functional outcomes and reduced medical complications (31, 32). Irrigation with warmed saline (37–40 °C) was shown to decrease recurrence compared to room-temperature fluid (33), whereas postoperative patient positioning (e.g., Trendelenburg) appeared to have minimal impact (34–36).

The role of corticosteroids remained debated: while the Dex-CSDH trial reported reduced reoperation rates with dexamethasone (37, 38), other studies found no significant benefit or raised concerns about side effects (39). Collectively, these trials reflected a shift toward minimally invasive, individualized, and multimodal CSDH management, integrating surgical optimization, endovascular intervention, and targeted medical therapy to improve outcomes and reduce recurrence.

## 4 Discussion

### 4.1 General information

This study presented a detailed scientometric analysis of 2010 publications on CSDH from the past 25 years in the WoScc, offering a comprehensive overview of this rapidly evolving field. The treatment paradigm for CSDH has shifted from traditional surgical drainage to an integrated “drug-intervention-surgery” model. Recent publication trends reflect this transition.

Between 2000 and 2010, research primarily focused on improving burr-hole drainage techniques and systems, as well as predicting etiological and prognostic factors. A key milestone during this period was the standardization of drainage protocols for cases with midline shift >1 cm, significantly improving surgical procedure consistency (20).

From 2011 to 2020, significant advancements occurred in minimally invasive and pharmacological treatment mechanisms. The anti-inflammatory mechanism of atorvastatin, which inhibits the NF-κB pathway, provided insights into CSDH pathogenesis and established pharmacological treatment as a crucial therapeutic modality (4, 40).

From 2021 to 2025, the development and implementation of minimally invasive intervention strategies marked the emergence of MMAE as a pivotal milestone in CSDH treatment (6, 22, 24). Concurrently, the clinical application of imaging prediction models and new embolic materials revitalized CSDH research, substantially transforming the traditional treatment paradigm (24, 26, 41, 42).

### 4.2 International research contributions

From the perspective of countries and institutions, the landscape of CSDH research reveals a distinct interplay between globally validated standards and region-specific innovations. The United States has consistently contributed to globally validated evidence through high-quality multinational trials. For instance, Harvard University led research involving 32 international centers (26). collaborative studies involving U.S., European, and Asian centers (e.g., the STEM trial) have demonstrated a universal reduction in hematoma recurrence rates with MMAE technology, establishing it as a standard care option across diverse healthcare systems (24).

Conversely, several significant contributions remain region-specific or are currently undergoing global validation. China has emerged as a leader in generating large-scale real-world data, particularly regarding the efficacy of atorvastatin. While multicenter RCTs within China have confirmed its benefits (4, 43), the generalizability of these findings to non-Asian populations requires further verification through ongoing international trials. Additionally, China’s dominant role in minimally invasive interventions (contributing >60% of global data in projects like MAGIC-MT and EMBOLISE) (6, 44) provides a massive sample size for protocol optimization, though the operational nuances may reflect regional surgical preferences. In Japan, the adjunctive use of traditional herbal medicine (*Goreisan*) combined with tranexamic acid represents a region-specific therapeutic strategy (27, 28), rooted in local pharmacological practices, which has not yet been widely adopted or validated in Western guidelines.

MMAE technology and operational protocol advancements were key research areas, with Europe and Japan focusing on evidence-based medicine and tech improvements. Notably, the British NICE guidelines adopted MMAE as a first-line treatment for CSDH.^3^

The CSDH research emphasized international collaboration, particularly between China and the U.S., showcasing technological complementarity and mutual standards recognition. In the MAGIC-MT and STEM studies, China contributed extensive sample data, while the U.S. optimized materials like the Squid liquid embolic agent, promoting MMAE as a key treatment standard (6, 24). However, a systematic review and meta-analysis revealed that combining MMAE with statin therapy did not significantly improve outcomes over MMAE alone in terms of complete resolution or recurrence rates (45).

### 4.3 Journal distribution and author contributions

Journal distribution closely reflected the progression of research hotspots. Core contributions were predominantly published in neurosurgery, interventional radiology, and comprehensive medical journals. *The Journal of Neurosurgery* and *Neurosurgery*, consistently emphasized innovations in surgical techniques, including advancements in burr-hole drainage (burr-hole craniostomy, twist-drill craniostomy) and endoscopic-assisted surgery (46). Since 2015, the *Journal of NeuroInterventional Surgery* has experienced a significant increase in CSDH-related publications, becoming the primary platform for MMAE research. This included developments in embolic materials and efficacy verification of combined surgical approaches. *The New England Journal of Medicine* published a series of high-quality RCTs, thereby advancing CSDH research to a new stage.

Highly cited authors displayed considerable diversity, reflecting the progression of research and treatment focus. Scholars investigating mechanistic exploration examined hematoma capsule neovascularization and the role of vascular endothelial growth factor (VEGF) (47). Technical innovation leaders advocated for MMAE procedure standardization, including embolic range grading and embolic material development (42, 48). Chinese researchers enhanced the international influence of MMAE and statin therapeutic protocols through multicenter studies and real-world data (4, 6).

### 4.4 Hotspots and development trends

The evolution of research hotspots in CSDH treatment exemplifies a paradigm shift from empirical management to precision medicine, a transition clearly mapped by citation bursts and co-citation clusters (49). This shift was elucidated through analysis of highly cited literature, keywords, and thematic trends. Between 2001 and 2010, the field focused on standardizing surgical procedures and elucidating pathological mechanisms. Pioneering work by Nakaguchi et al. (50) established CT-based classification system, correlating imaging features with recurrence risk (51). Since 2005, controversies surrounding surgical techniques highlighted unresolved clinical questions, including surgery timing, number of burr-holes, drainage location, and irrigation use (20, 52–55). Concurrently, research on anticoagulation demonstrated that antiplatelet drugs increased the risk of CSDH (13, 56–59). Keywords such as “recurrence rate” (58, 60), “closed-system drainage” (61, 62) and “twist drill craniostomy” (16, 63) emerged as predominant themes.

From 2011 to 2020, drug treatment saw significant progress. In 2016, a pivotal RCT study involving atorvastatin demonstrated that pharmacological intervention could reduce hematoma volume and decrease the necessity for surgical intervention, thereby challenging the traditional surgery-based treatment paradigm (4, 40, 64). By 2019, the use of MMAE highlighted the advancement of interventional technology. During this time, key terms like “atorvastatin” (65), “inflammatory factors” (66, 67), “embolism” (68, 69), and “imaging evaluation” (70, 71) became important, steering research towards drug intervention combinations.

Since 2021, research has focused more on minimally invasive and precision treatments. The 2024 MAGIC-MT study confirmed interventional therapy as the primary treatment (6). Studies on “MMAE” and “liquid embolic materials” continue to advance personalized and intelligent methods (5, 42). The recent use of 3D-T2-FLAIR MRI has clarified the link between meningeal lymphatic drainage and recurrence (72, 73).

### 4.5 Clinical trial landscapes

CSDH clinical research has undergone substantial evolution, shifting toward multidimensional management strategies beyond conventional surgical techniques. Unilateral burr-hole drainage has increasingly replaced bilateral approaches due to its procedural simplicity and reduced invasiveness, with no significant differences in recurrence rates observed between the two methods across multiple studies (12–14).

Subperiosteal drainage has demonstrated a clinically meaningful reduction in recurrence rates (10–15%) compared to traditional subdural drainage, particularly benefiting elderly patients who often face higher recurrence risks (18, 20, 21). For bilateral CSDH management, retrospective analyses of 57 cases revealed that not all patients require bilateral procedures; unilateral drainage sufficed for many, as bilateral cases treated with unilateral surgery exhibited comparable midline shift distances and recurrence rates to those undergoing bilateral drainage (*p* > 0.05), though bilateral cases presented greater average hematoma thickness (*p* < 0.01) (23).

Recurrence prevention remains a pivotal challenge, with evidence of stratification becoming increasingly important. Globally validated evidence now supports MMAE as a transformative adjunct, with multiple multinational RCTs confirming its ability to reduce recurrence rates from 20–30% to below 10% without significant safety concerns across different ethnic groups (6, 74, 75).

Certain preventive measures exhibit region-specific validity. For example, the synergistic effect of *Goreisan* (a Japanese herbal medicine) with tranexamic acid is well-documented in Japanese cohorts (27, 28, 76), but lacks validation in large-scale Western populations. For special populations, anticoagulated patients (e.g., on aspirin) may safely continue therapy perioperatively without elevated bleeding risk (77). Furthermore, while corticosteroids show efficacy, their benefit appears to be subtype-specific and potentially influenced by regional differences in patient inflammatory profiles and comorbidities (78, 79).

Postoperative management has been refined through rigorous optimization. The DRAIN TIME 2 multicenter trial (27) demonstrated that shortening drainage duration from 72 to 24–48 h maintained comparable recurrence rates while reducing hospital stays by 1.69 days. Early functional mobilization initiated within 24–48 h postoperatively (GET-UP study) significantly improved neurological outcomes and reduced complications (31, 32), and warm irrigation fluid (37–40 °C) lowered recurrence rates by enhancing local microcirculation (33, 80, 81). Additionally, early drainage cessation (within 24–48 h) was not associated with increased recurrence risk (82), supporting streamlined protocols.

### 4.6 Pathophysiological and therapeutic shift

Pathophysiological insights have deepened the mechanistic understanding of CSDH. Gardner’s 1932 “osmotic gradient and semipermeable membrane” theory (36) explains progressive expansion. This theory is now complemented by evidence linking CSDH to post-traumatic subdural effusion evolution, elevated IL-6/IL-8 levels, aquaporin (AQP) channel activation, fibrinolysis hyperactivity (t-PA-driven plasminogen conversion), and hematoma membrane neovascularization (83). These mechanisms clarify the typical 3-week symptom latency post-trauma and continuous expansion.

The therapeutic paradigm is advancing toward personalized precision medicine. Treatment strategies now integrate hematoma size, patient age, and comorbidities for tailored approaches (79, 84, 85), while MMAE is transitioning from adjunctive research to standard care (6, 74). Emerging synergies, such as MMAE combined with corticosteroids or Goreisan (76), and AI-driven applications in clinical trial design and outcome prediction (86), imply the next frontier. Collectively, these evidence-based advances have substantially elevated cure rates (e.g., ~90% with statin therapy in large cohorts (87)) and quality of life, establishing a robust foundation for contemporary CSDH management.

The evolving focus of research was driven by several factors, including the aging population, the widespread use of antithrombotic drugs (55), advancements in imaging technology (70, 88), and the identification of biomarkers (such as IL-6 and VEGF as potential targets) (89–91). Future research is anticipated to expand into various domains, including genomics (e.g., neutrophil extracellular traps formation in the dural and hematoma membranes might be involved in the pathogenesis of CSDH) (90), artificial intelligence (e.g., the clinical application of multimodal imaging prediction models) (92, 93), novel materials (e.g., biodegradable embolic materials to mitigate complications) (94), and global collaboration (e.g., the development of an international multicenter trials) (48). These efforts aim to ultimately enhance the precise management of the entire course of CSDH.

### 4.7 Limitations

This study has several limitations. Firstly, reliance on WoScc for structured metadata may introduce language bias by excluding non-English publications, despite also using PubMed data. Secondly, citation-based indicators tend to favor older articles, possibly underestimating recent studies’ impact. To address this, we included keyword burst detection and trend mapping to identify emerging research. Lastly, while PubMed offers valuable clinical trial data, its integration into network-based scientometric analyses is limited compared to WoScc, potentially restricting the depth of clinical insights in co-citation and collaboration networks.

## 5 Conclusion

This bibliometric analysis of research on CSDH spanning the years 2001 to 2025 highlighted a field in dynamic evolution, marked by consistent growth in research activity, increased geographic multicenter collaborations, and a shift in thematic priorities. CSDH research has experienced three transitions: an initial emphasis on the surgical procedure standardization and pathological mechanisms exploration; subsequent advancements in pharmacological interventions and minimally invasive therapies; and a recent shift toward individualized management informed by omics technologies and biomarkers. Future research should prioritize genomic signatures, biocompatible embolic material innovation, and optimization of cross-regional clinical collaborations. These efforts are crucial for addressing unmet clinical needs and advancing CSDH treatment toward more precise, equitable, and globally applicable therapeutic paradigms.

## Data Availability

The original contributions presented in the study are included in the article/Supplementary material, further inquiries can be directed to the corresponding authors.

## Footnotes

1
https://cnsknowall.com

2
https://www.chiplot.online

3
https://www.nice.org.uk/guidance/ipg779
