# Supplementary material for: From burr-hole to embolization: unveiling the evolution of chronic subdural hematoma research (2001–2025)
Source: Front Neurol. 2026 Jun 12;17:1780300. doi: 10.3389/fneur.2026.1780300 (PMC13303121; doi:10.3389/fneur.2026.1780300)
Supplement: Supplementary file 2 [file Table_1.DOCX]

**Supplementary File 1. Search strategies and retrieval formula for WOScc**

((TS=("chronic subdural hematom*" OR "chronic subdural haematom*" OR CSDH)

NOT

(TS=("acute subdural" OR "traumatic brain injury" OR "animal model" OR rat OR mice OR "Commission on the Social Determinants of Health"))) AND LA=(English))

Document Types: Article or Review Article or Meeting Abstract or Early Access or Proceeding Paper

**Search strategies and retrieval formula for PubMed**

((("hematoma, subdural, chronic"[MeSH Terms] OR ("chronic subdural hematoma*"[Title/Abstract] OR "chronic subdural haematoma*"[Title/Abstract] OR "CSDH"[Title/Abstract])) NOT (("hematoma, subdural, acute"[MeSH Terms] OR "brain injuries, traumatic"[MeSH Terms] OR "acute subdural"[Title/Abstract] OR "traumatic brain injury"[Title/Abstract] OR "animal model*"[Title/Abstract] OR "rat"[Title/Abstract] OR "mice"[Title/Abstract] OR "Animals"[MeSH Terms]) NOT "Humans"[MeSH Terms])) AND "English"[Language] AND "Humans"[MeSH Terms]) AND ((clinicaltrial[Filter]) AND (2001:2025[pdat]))
